# Bronchitis

**Published:** 1878-05

**Authors:** 


*Bronchitis* is a disease of the branches of the windpipe, which are the tubes, conveying the air from the windpipe to the lungs themselves. The distinguishing feature of Bronchitis, as above stated, is a stuffed up feeling. The eyes and nose water very much. There is a sensation of oppression. In fact, Bronchitis is a common cold, lasting for many days. But custom has given the name of "*Bronchitis*" to the symptoms of a common cold when they have become permanent. Properly speaking, this is *chronic bronchitis*, for shortness called "*bronchitis.*" The reader will do well to remember that "*bronchitis*," that puzzling name, that mysterious, that fondly hugged designation, pronounced so often, so glibly and familiarly by the deluded consumptive, is a common cold protracted. And as in a common cold the cough is not the first symptom, not appearing sometimes for a day or two, and then becomes the main feature: so the first symptoms of bronchitis are as above stated, but they end in a cough, which soon becomes the all-absorbing symptom, tearing and racking the lungs day and night, with scarcely any intermission sometimes, and in this, is strikingly different from consumption, for its cough is mainly at night and in the morning.

While, then, throat-ail is an affection of the voice-making organs, and croup is located in the windpipe, and bronchitis belongs to the air-tubes, which come out from the windpipe as the branches of a tree come out from its body, diverging widely; so consumption is a disease which attacks the lungs themselves, answering to the leaves of the tree, the lungs being at the extreme points of the air-tubes, as leaves are at the extremities of the branches of a tree.

But it is of consumption that these pages mainly speak. No doubt some degree of minuteness will be acceptable to the great mass of readers.

Imagine each leaf of a tree to be a small bladder or air-cell, filled with air, reaching them through the branches which draw their supply from what passes along the windpipe derived from without. These air-cells are of various sizes, from a pin head to a pea, and thinner than any paper we know of. All over these air-cells, like a vine on a wall, there are branches of bloodvessels, bringing the blood directly from the heart. These blood-vessels must necessarily be very minute, and to pass along them with any degree of facility the blood must necessarily be very pure, that is, it must not be thick, must not have in it foreign matter, sediments, the wastes of the system, its impurities. Mush will not readily flow through a hose-pipe. If water was so filled with mud as to have the consistency of stirabout, all the efforts of our gallant firemen would be in vain. This idea is of such vital importance, theoretically and practically, that the reader is earnestly desired, before he proceeds farther, to master it fully.

Of not less importance is it to remember a familiar fact that if a hose-pipe lays in a direct line, the water passes along with great ease and power, but if the hose are laid crooked and angular, even the purest water moves slowly.

If you take a common bladder, fully distended with air and draw straight lines from its neck to the bottom, those lines will become very crooked, if a great portion of the air be allowed to escape.

In health, the lungs are fully distended. The blood vessels are, comparatively speaking, in a direct line. The blood itself is pure, and from both causes it courses along the channels of life with rapidity and ease.

